# Longitudinal functional outcomes and late effects of radiation following treatment of nasopharyngeal carcinoma: secondary analysis of a prospective cohort study

**DOI:** 10.1186/s40463-022-00593-7

**Published:** 2022-11-08

**Authors:** Gia Gill, Ciaran Lane, Candace Myers, Evan D. Kerr, Pascal Lambert, Andrew Cooke, Paul D. Kerr

**Affiliations:** 1 Department of Otolaryngology-Head and Neck Surgery, Max Rady College of Medicine, University of Manitoba, GB421 - 820 Sherbrook Street, Winnipeg, MB R3A 1R9 Canada; 2 Speech Language Pathology, Department of Psychosocial Oncology, CancerCare Manitoba, Room ON1018, 675 McDermot Ave, Winnipeg, MB R3E 0V9 Canada; 3 Max Rady College of Medicine, University of Manitoba, 750 Bannatyne Ave, Winnipeg, MB R3E 0W2 Canada; 4 Department of Epidemiology, CancerCare Manitoba, Room ON-2114, 675 McDermot Ave, Winnipeg, MB R3E 0V9 Canada; 5 CancerCare Research Institute, CancerCare Manitoba, CancerCare Manitoba Research Institute Office, 675 McDermot Ave, Winnipeg, MB R4E 0V9 Canada; 6 Section of Radiation Oncology, Department of Radiology, Max Rady College of Medicine, University of Manitoba, Room GA216, 820 Sherbrook Street, Winnipeg, MB R3T 2N2 Canada; 7 Radiation Oncology, CancerCare Manitoba, 820 Sherbrook Street GA216, Winnipeg, MB R3T 2N2 Canada

**Keywords:** Nasopharyngeal carcinoma, Functional outcomes, IMRT

## Abstract

**Background:**

The study objectives were: provide longitudinal data on upper aerodigestive tract function and late complications following IMRT for nasopharyngeal carcinoma, and elucidate factors that might predict a worse outcome. The hypotheses were: (1) Despite advances such as IMRT, radiation will cause significant functional decline and late complications that often progress or arise years after treatment. (2) Larger radiation volume will be associated with poorer outcomes.

**Methods:**

Longitudinal, observational cohort study of nasopharyngeal carcinoma patients with retrospective analysis of prospectively collected, population-based data. Late sequelae and validated measures of overall performance, speech, and swallowing were documented pre-treatment and 3,6,12, 24, 36 and ≥ 60-months post-treatment.

**Results:**

Forty-two patients treated curatively with radiation (N = 9) or chemoradiation (N = 33) were followed for a median 74 months. Functional outcomes showed an initial nadir at 3 months associated with acute effects of treatment, followed by initial recovery. There was subsequent functional decline years post-treatment with advancing dysphagia/aspiration, trismus, muscle spasm, and hypoglossal nerve palsy. Univariable regression analysis revealed that increasing high-dose radiation volumes (PTV 70 Gy) were associated with increased likelihood of less than solid diet (Performance Status Scale (PSS)—Normalcy of Diet score < 50; *p* = 0.04), and reduced PSS—Understandability of Speech (*p* = 0.005). The probability of poor outcome increased with time. Eleven percent of patients were tube feed dependent at ≥ 5 years.

**Conclusions:**

Despite improvements in radiation delivery, late effects of radiation remain common. Higher radiation volumes are associated with poorer outcomes that worsen over time.

**Graphical Abstract:**

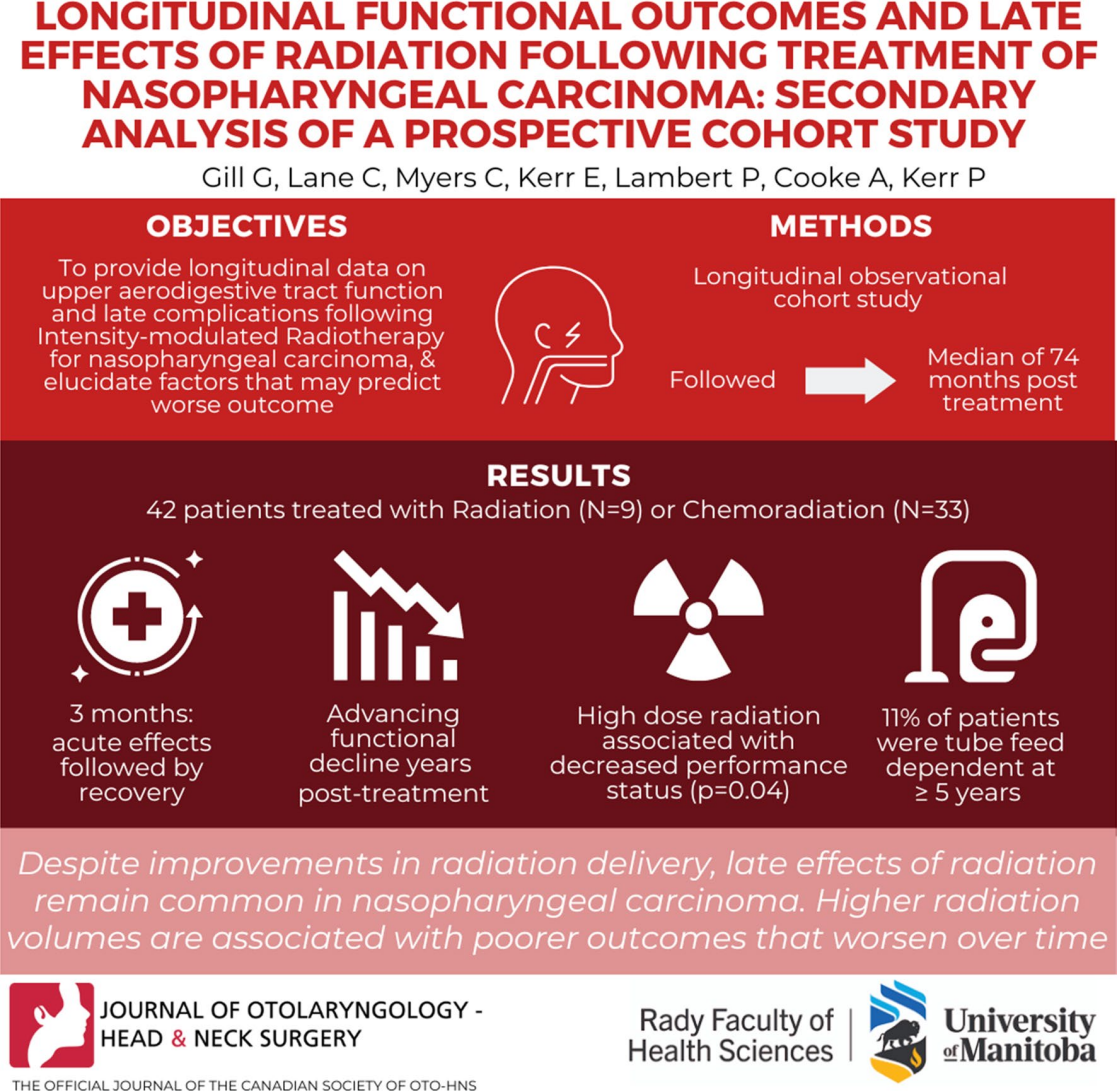

**Supplementary Information:**

The online version contains supplementary material available at 10.1186/s40463-022-00593-7.

## Background

Intensity-modulated radiotherapy (IMRT) has replaced the use of conventional radiotherapy for treatment of nasopharyngeal carcinoma (NPC) [1]. IMRT is an advanced form of 3-dimensional conformal RT that delivers a dose of radiation to the target tumour while reducing irradiation to surrounding tissues, mitigating some negative effects of radiation [2]. Despite the use of IMRT, patients still suffer functional decline following treatment of NPC. The time-course, nature and prevalence of these late radiation effects have not been well delineated in the literature. Published reports on functional outcomes and late toxicities are often selective, have short follow-up, or combine multiple subsites of head and neck cancer. The objective of this study was to provide comprehensive, longitudinal data on upper aerodigestive tract function and late complications following IMRT for nasopharyngeal carcinoma, and to elucidate factors that might predict a worse outcome. The hypotheses were: (1) Despite IMRT, radiation still frequently causes significant functional decline and late complications that often progress or arise many years after treatment. (2) Important predictors of increased late effects include advanced age, advanced stage, higher radiation volume, and the addition of chemotherapy.

## Methods and materials

### Study design

The design of this study was a population-based, prospectively collected, retrospectively reviewed cohort study. Participants were recruited from the Manitoba Cancer Register, which is a comprehensive and accredited population-based registry [3]. Participants were recruited consecutively, completing supervised self-reported questionnaires and clinical assessment by a speech and language pathologist (SLP) at 3, 6, 12, 24, 36, and ≥ 60 months post-treatment appointments. In the first year, assessments could vary by as much as ± 1 month. At 24 and 36 months visits could vary ± 3 months. Follow up in the ≥ 60 month period ranged from 5 to 12 years. Acquisition of this data was part of the regular clinical follow-up of all patients treated for upper aerodigestive tract cancer within CancerCare Manitoba, the solitary tertiary cancer centre for the Province of Manitoba, Canada. The study was reviewed and approved by the Research Ethics Board, Office of Research Ethics and Compliance, University of Manitoba (Approval #HS19561). This study was exempt from individual-level consent as patients were receiving standard of care therapy and this was considered a low-risk, noninterventional, observational study.

Pre-treatment was defined as prior to initial radiation. Post-treatment was defined as the period following the last day of radiation. Electronic medical records were reviewed retrospectively for demographic, oncologic, selected functional outcome, and late effects (complications of radiation) data for patients treated between 2008 and 2016 inclusive. Patients were included in this study if they had American Joint Committee on Cancer (AJCC VII) stage I-IV (M0) nasopharyngeal carcinoma and were treated with curative intent with IMRT alone or with chemo-radiation. Patients were excluded if they underwent previous head and neck irradiation to the primary tumour, palliative intent defined by a radiation dose < 50 Gy, or had a previous head and neck cancer within 5 years prior to diagnosis.

Data from patients who suffered recurrent disease was censored at the time of the recurrence. All patients were treated within the same tertiary care, academic health network: CancerCare Manitoba and the University of Manitoba teaching hospitals. All patients had their management reviewed at the multidisciplinary case conference of the Head and Neck Disease Site Group.

The Manitoba Cancer Registry (MCR) is a comprehensive and accredited population-based registry for the province of Manitoba, Canada; population 1.2 million. The MCR is a member of the North American Associated of Central Cancer Registries which administers a program that review member registries for their ability to produce complete, accurate, and timely data. Fields included diagnoses coded using the International Classification of Diseases 10th revision for Canada (ICD-10-CA), age, and stage. Health care is freely provided by the government and non-participation in the plan is rare. Therefore, the registry and any derived cohort can be considered complete and population based. All incident registry cases of nasopharyngeal carcinoma from 2008 to 2016 inclusive were reviewed to ensure all eligible cases were included.

### Functional outcomes protocol

CancerCare Manitoba established prospective collection of selected functional outcomes following treatment of head and neck cancer as part of routine follow up in 2003. The protocol was intended to capture functional outcomes consistent with the International Classification of Functioning, Disability and Health (ICF) [4, 5]. The outcomes chosen for this study included 7 instruments which have proven inter-rater reliability and validity and are widely used in head and neck cancer patient assessments.

Two validated clinician-rated general performance status scales were included: The Karnofsky Performance Status (KPS) scale and the Eastern Cooperative Oncology Group (ECOG) toxicity and response criteria scale are standard tools used to assess performance in activities of daily living [6].

Five validated clinician-rated scales of swallowing and communication were included: The Performance Status Scale for Head and Neck Cancer Patients (PSS-HN) which describes performance on three subscales: normalcy of diet (maximum oral diet texture tolerated), eating in public, and understandability of speech; and the Royal Brisbane Hospital Outcome Measure for Swallowing (RBHOMS) which is a measure of swallowing and tube feed dependency [7].

To provide clinical context to the reader, a brief summary of the 3 PSS-HN scales are as follows: (1) “Normalcy of Diet” is a diet texture scale in increments of 10, from 0 to 100. Score 0: NPO; 10–30 = liquid or pureed diet; 40–50 = soft diet; 60–90 = increasingly solid diet but some limitations; 100 = normal diet, no restrictions. (2) “Understandability of Speech” is scaled in increments of 25, from 0–100. Score 0 = never understood; 25 = difficult to understand; 50 = usually understood with face-to-face conversation; 75 = usually understood in all circumstances; 100 = always understood. (3) “Eating in Public” is scaled in increments of 25, from 0 to 100. Score 0 = always eat alone; 25 = only eats at home in presence of selected people; 50 = eats in front of selected people in selected locations; 75 = no restriction to place, but modified diet; 100 = no restrictions to diet or place [8].

The validated clinician-rated Royal Brisbane Hospital Outcome Measure for Swallowing (RBHOMS) was used to evaluate relative feeding tube dependency. This 10-point scale measures relative feeding tube dependence and dysphagia, with higher scores indicating better function. RBHOMS scores of 1–5 indicate that the patient is tube feed dependent, either totally (i.e. patient NPO; scores 1–3) or partially (scores 4–5). RBHOMS scores of 6–10 indicate that the patient can sustain themselves nutritionally and is not feeding tube dependent. A RBHOMS scores of 6–7 cover liquid diets to introduction of solid foods; a score of 8 indicates that solids have become a consistent part of the diet but the diet is still substantially modified, 9 indicates diet is near normal solid diet with minor modifications [9].

Finally, the Voice Handicap Index-10 (VHI-10) was used [10]. This patient-rated scale has 10 questions with answers ranging from 0 (never) to 4 (always) for a total of 40 possible points with a higher score indicating worse self-perceived voice handicap. The VHI-10 has been collapsed into 3 ranges, 0–9 (never to almost never severe), 10–19 (sometimes severe), and > 20 (more than sometimes severe). Ranges are based on clinically relevant points in functional outcome scales for analysis, limited by the low number of patients with scores > 20.

In addition to tracking functional outcomes, other late effects of treatment were documented as part of the scheduled pre- and post-treatment SLP visits. The validated modified Edmonton Symptom Assessment Scale (mESAS) is a patient-rated visual analog scale used to measure the severity of multiple symptoms of advanced cancer. Symptoms are scored from 0 to 10 with a score of 10 indicating the worst possible symptoms. The mESAS was used to collect the patient-reported symptom of xerostomia and dysphagia [11]. SLP also documented the presence of the following problems: trismus; neck flexion deformity; cranial nerve 12 palsy; whether dysphagia was bad enough to require a videofluoroscopic swallowing study in the preceding interval, and whether aspiration was evident; treatment for aspiration pneumonia; presence of muscle spasms; presence of tracheostomy; and feeding tube dependency.

Patient demographic and oncologic data collection included age, gender, AJCC (7th Edition) Prognostic Stage, T classification, N classification, radiation dose and volume, chemotherapy details, date and location of recurrence, and date and cause of death. Radiation volumes collected included: Gross tumour volume (GTVp); gross nodal volume (GTVn); planning target volume receiving at least 70 Gy (PTV70); planning target volume receiving at least 59 Gy (PTV59); and sum of all target volumes receiving a therapeutic dose (PTVall); and mean dose to left and right parotid (MD parotid L,R).

### Statistical analysis

Overall survival (OS) and disease-free survival (DFS) were determined using standard Kaplan–Meier methodology, whereas disease-specific survival (DSS) was determined using cumulative incidence with competing risk for death. Times for assessments were measured from the date of the last radiation treatment. Patients with recurrence were followed and assessed until death or end of study follow-up. Patients were censored at the time of death, with or without recurrence. There were two components to the late effects analysis: (1) A descriptive analysis of function and side effects over time focusing on the nature and severity of late effects and whether there was a trend to worsening function and side effects in later time intervals, and (2) A regression analysis of factors that predict poor functional outcome.

Regression models were used to predict outcomes and examine the relationship between baseline predictors and outcomes. Logistic mixed models were used to predict binary outcomes with repeated measurements, and the output was marginalized. Quantile regression with a clustered variance was used to predict ordinal outcomes with repeated measurements and reported median differences. Regression models analyzed data from 1 year and onward, which excluded the acute post-treatment effects. Regression models were used to examine the association between predictors and outcomes, as well as provide estimated marginalized probabilities (for binary outcomes) and predicted median values (ordinal outcomes) over time to describe functional health during follow-up. The relationship between continuous predictors and outcomes were demonstrated with predicted values over time for the 10th, 50th, and 90th percentile values of the continuous predictor. Natural splines were used to account for non-linear time effects in regression models. R version 4.03 was used to analyze the data with the following packages: cmprsk, GLMMadaptive, quantreg, splines, and survival.

## Results

Ninety-seven patients were assessed for NPC in Manitoba from 2008 to 2016. Fifty-five of the original 97 patients diagnosed with NPC were excluded because they were treated palliatively, did not complete treatment, or declined regular participation in all aspects of the study. There were 42 patients included in the study who were treated curatively with radiation (N = 9) or chemoradiation (N = 33), and who participated in regular follow-up with SLP, contributing to the full array of clinician-rated and patient-rated functional outcome surveys. Patients were censored at the time of recurrence or death (N = 7) or when lost to follow-up (N = 7 (17%), all > 3 years post-treatment). Data was available for all 42 patients at one year, 41/42 (98%) at 2 years, 38/42 (90%) at 3 years, and 28/42 (67%) at ≥ 5 years.

Patient and treatment characteristics are shown in Table 1. The mean age was 52 years. Median follow up was 72 months. At 5 years, the overall survival (OS) was 87.6% and disease free survival (DFS) was 82.9% consistent with high rates of disease control seen in the literature.

**Table 1:** Description of cohort

Description of cohort (N = 42)			
		N	%
Age	Mean (SD)	52.4	(14.4)
Gender	Female	16	38.1
	Male	26	61.9
T stage	I	15	35.7
	II	10	23.8
	III	6	14.3
	IV	11	26.2
N stage	0	14	33.3
	I	11	26.2
	II	13	31.0
	III	4	9.5
M stage	0	39	92.9
	X	3	7.1
AJCC stage	I	4	9.5
	II	10	23.8
	III	12	28.6
	IV	16	38.1
GTVp volume (mL)	Median (Q1–Q3)	25.8	(12.6–47.7)
PTV 70 Gy (mL)	Median (Q1–Q3)	210.6	(115.6–330.8)
PTV all (mL)	Median (Q1–Q3)	808.6	(686.8–1022.1)
MD parotid L (mL)	Median (Q1–Q3)	3598	(3242–4222)
MD parotid R (mL)	Median (Q1–Q3)	3564	(3308–3859)
MD parotid L + R/2 (mL)	Median (Q1–Q3)	3630	(3322–4156)
Treatment	Chemoradiation	33	78.6
	Radiation	9	21.4
Radiation dose	5400	1	2.4
	5940	1	2.4
	7000	39	92.9
	Missing	1	2.4

### Longitudinal functional outcomes and late complications of treatment

**Fig. 1:**
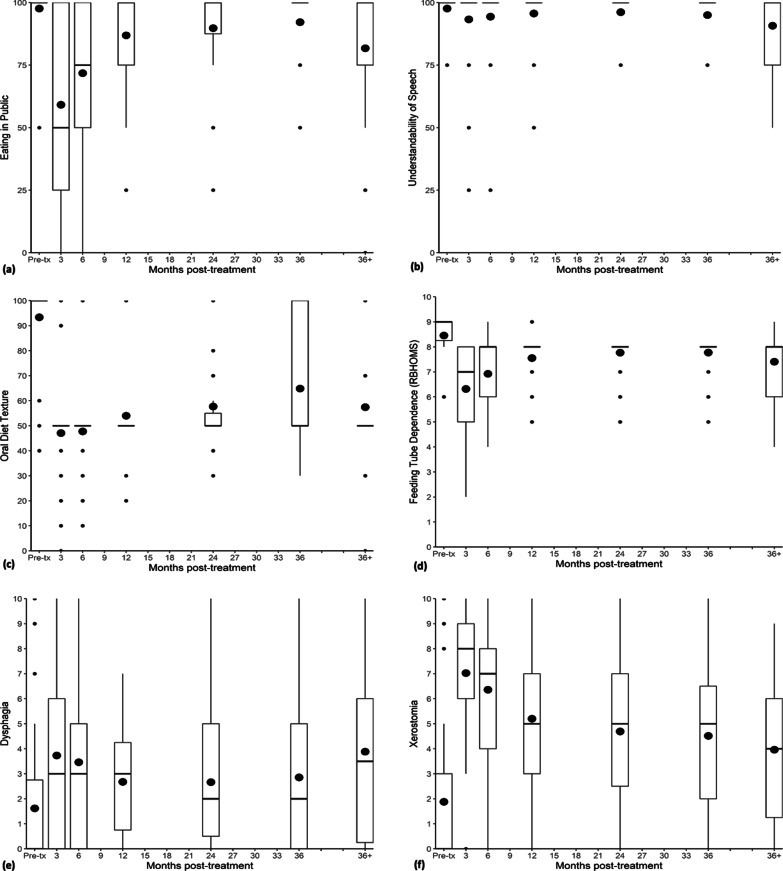
**a** Eating in Public (PSS-HN) over time. **b** Understandability of Speech (PSS-HN) over time. **c** Oral Diet Texture (PSS-HN) over time. **d** Royal Brisbane Hospital Outcome Measure of Swallowing (RBHOMS) over time. **e** Dysphagia outcomes from the modified Edmonton Symptom Assessment Scale (mESAS). **f** Xerostomia outcomes from the modified Edmonton Symptom Assessment Scale (mESAS)

**Fig. 2:**
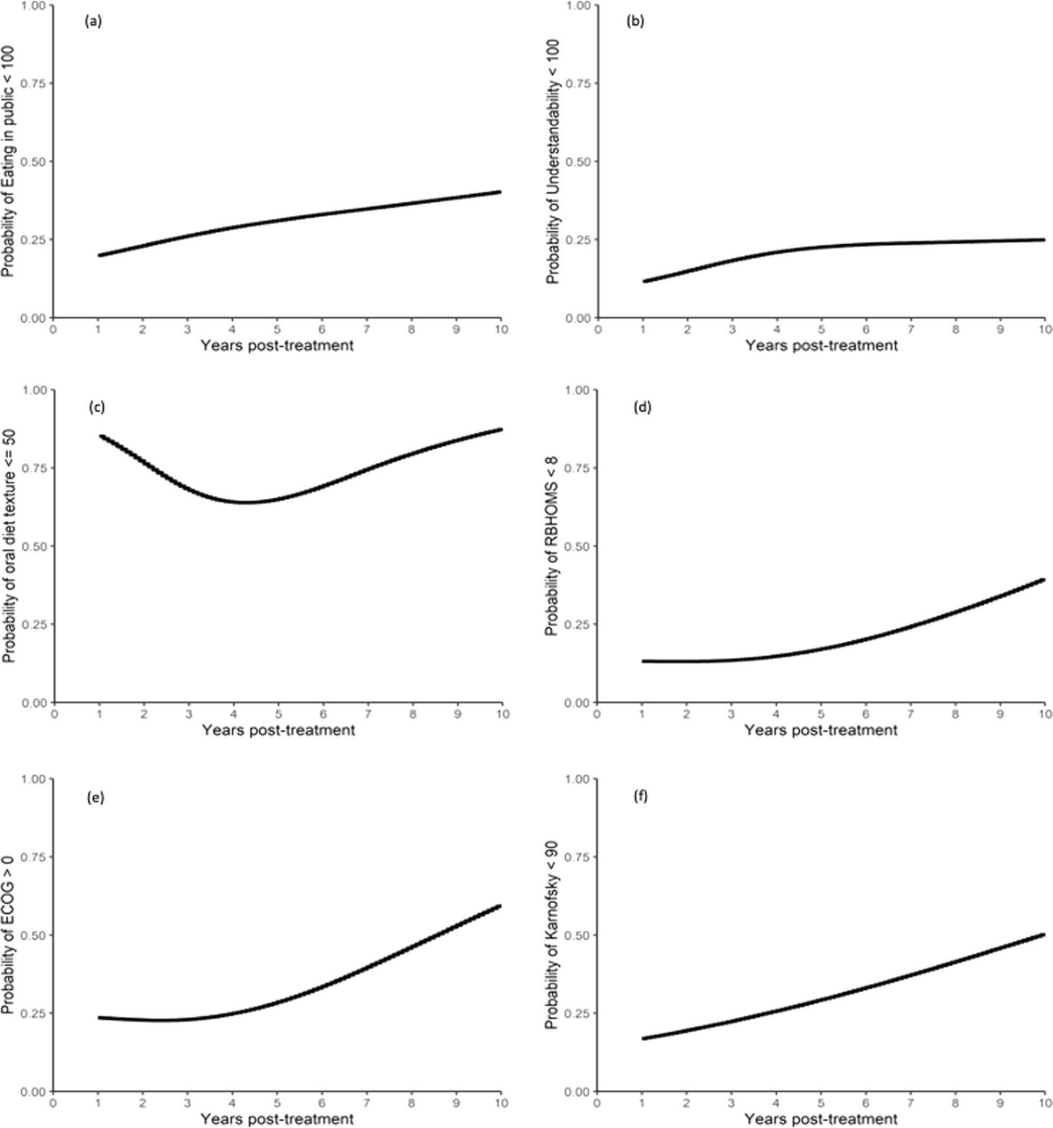
**a** Probability plot of Eating in Public (PSS-HN) over time from using regression analysis. **b** Probability plot of Understandability of Speech (PSS-HN) over time from using regression analysis. **c** Probability plot of Oral Diet Texture (PSS-HN) over time from using regression analysis. **d** Probability plot of Royal Brisbane Hospital Outcome Measure of Swallowing (RBHOMS) over time from using regression analysis. **e** Probability plot of Eastern Cooperative Oncology Group (ECOG) toxicity response criteria scale over time from using regression analysis. **f** Probability plot of Karnofsky Performance Status (KPS) scale over time from using regression analysis

Longitudinal patient-rated outcomes from PSS-HN, RBHOMS, and ESAS dysphagia and xerostomia data are displayed in the box plots (Fig. 1a–f). The large dots added to each of these plots indicate the mean. Several of these plots demonstrate the anticipated early decline in function consistent with the acute effects of treatment followed by a period of functional improvement as patients recovered from these acute effects. Subsequently, some of these plots suggest functional decline in the later time periods. To further investigate these potential late effects, regression analysis was used to generate 5-year estimates of reduced functional outcomes which can be seen in Table 2. The same analysis was used to generate probability plots of function over time to better delineate trends in function over time. These can be seen in Fig. 2a–f, which depict the likelihood of any reduction of a patient’s ability to eat in public (PSS-HN Eating in Public score < 100; Fig. 2a); the likelihood of any reduction to a patient’s ability to be understood (PSS-HN Understandability of Speech score < 100; Fig. 2b); the likelihood of patients being unable to swallow anything more than a soft diet (PSS-HN Normalcy of Diet score ≤ 50; Fig. 2c); the likelihood of a patient requiring tube feeding or having a diet that is very limited in solid food intake (RBHOMS score < 8; Fig. 2d); or having less than the highest Karnofsky and ECOG scores (Figs. 2e–f).

**Table 2:** 5 year estimates of reduced functional outcomes calculated using regression analysis

		5-year estimate (produced from regression models)
Eating in public	Probability of under 100 (restricts in public and lower)	31%
Understandability of speech	Probability of under 100 (most of the time or less)	23%
Oral diet texture	Probability of 50 and under (soft chewable foods to liquids)	65%
RBHOMS	Probability of under 8 (modified diet to total dependence)	17%
Voice Handicap Index 10	probability of above 0 (1–40)	39%
Karnofsky	Probability of under 90 (some signs to unable to do active work)	29%
ECOG	Probability of above 0 (restrictions to unable to do active work)	29%
Dysphagia	Median	2.18
Xerostomia	Median	3.84

Eleven percent of patients (3/28) who were still in the study and followed > 5 years were tube feed dependent at last follow up. One patient was both tracheostomy and gastrostomy dependent, and likely died of aspiration pneumonia greater than 5 years post-treatment.

**Fig. 3:**
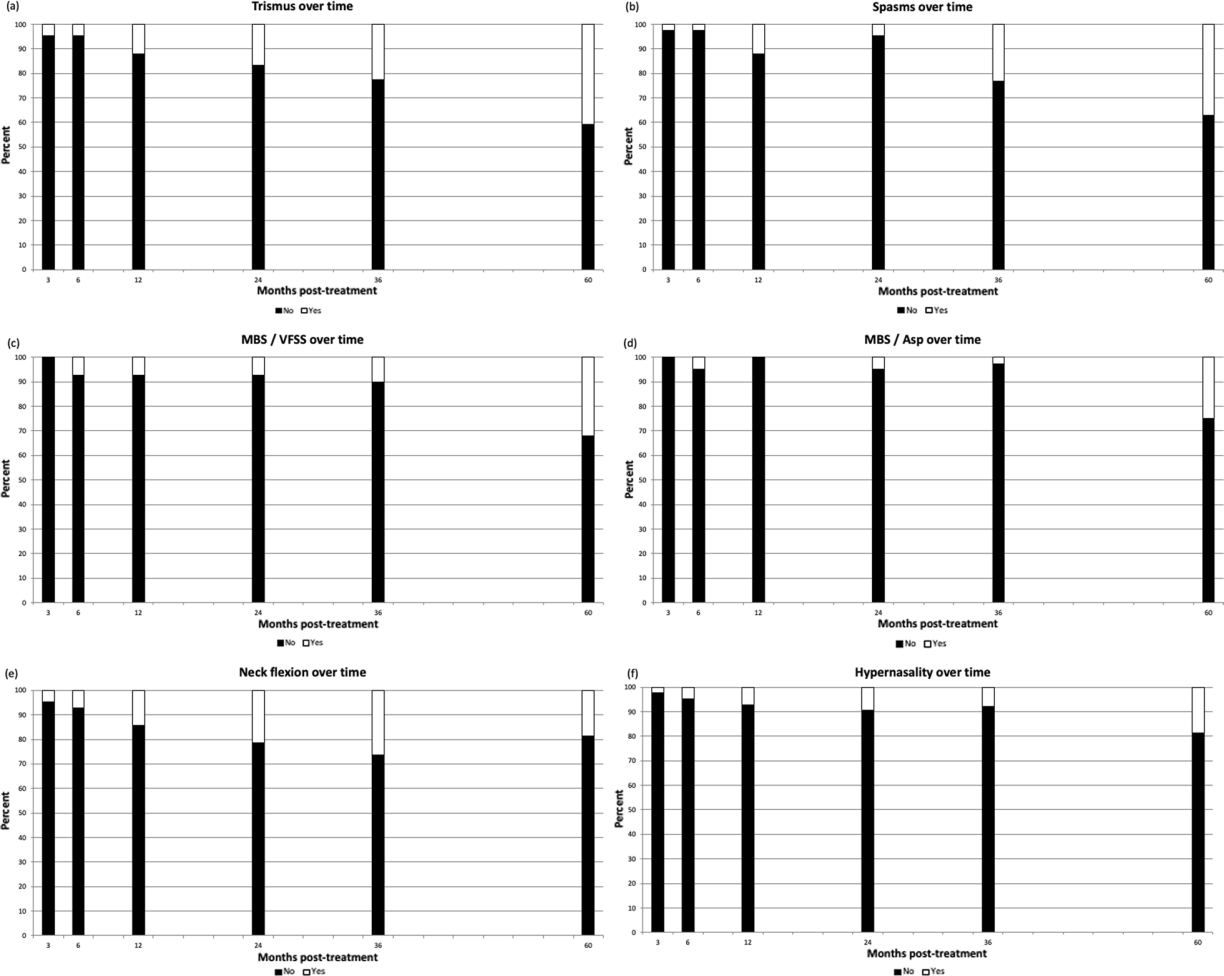
**a** Trismus rates over time. **b** Muscle spasm rates over time. **c** Rates of aspiration on VFSS over time. **d** Aspiration pneumonia rates over time. **e** Rates of neck flexion deformity over time. **f** Rates of hypernasality over time

Longitudinal clinician-rated late effects are depicted in the bar graphs (Fig. 3a–f). The percentage of patients with trismus (Fig. 3a), recurrent muscle spasms (Fig. 3b), swallowing troubles bad enough to require videofluoroscopic swallow study or modified barium swallow (VFSS/MBS; Fig. 3c), demonstrating aspiration on VFSS (MBS Aspiration; Fig. 3d), and demonstrating hypernasal speech (Fig. 3f) are higher in the ≥ 5 year time period. Neck flexion deformity is a common issue (Fig. 3e). There was tongue hemiparesis suspected in 5/28 (18%) of patients assessed in the ≥ 5 year time period (not shown in figures).

### Regression analysis of factors predicting poor outcome

Regression analysis was used to assess the degree to which age, gender, overall stage, radiation volume, and the addition of chemotherapy could predict poor outcomes (Regression tables are only available in the Additional file 1: Table S3—Appendix 2; provided for the reviewers’ information). Limited numbers precluded including more than follow-up time and an additional predictor to a model. Increasing radiation volumes were associated with poor speech and swallowing outcomes: Larger PTV 70 Gy was associated with increased likelihood of less that solid diet (Normalcy of Diet score < 50; *p* = 0.04), and increases in any radiation volumes (PTV 70 Gy, GTVp, and PTVall) were associated with reduced understandability of speech (*p* = 0.003–0.005). Advanced overall stage were associated with Normalcy of Diet scores < 50 (*p* = 0.01). Advanced age was associated with increased likelihood of having Karnofsky < 90 (*p* = 0.04), and ECOG > 0 (*p* = 0.05). Gender and addition of systemic therapy were not associated with poor outcomes, but it is important to note that only 9 patients did not have chemotherapy, so the study was not powered adequately to detect differences between patients treated with and without adjuvant systemic therapy.

**Fig. 4:**
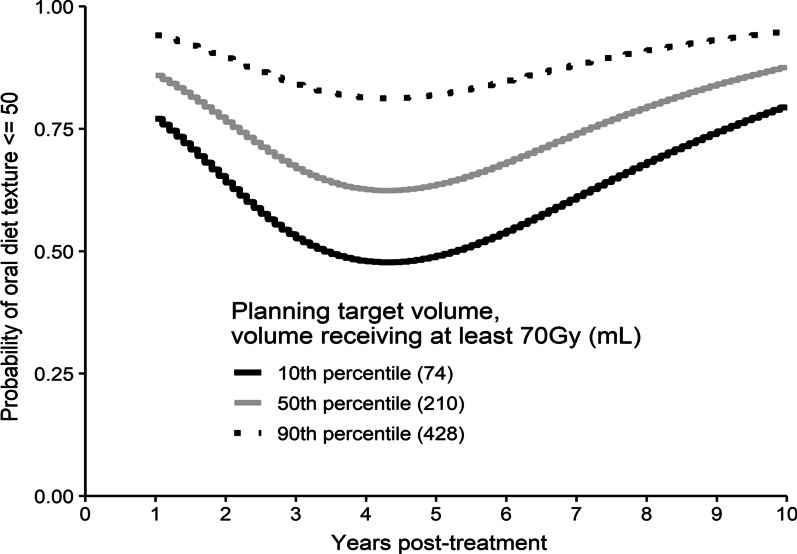
Probability curves over time showing the likelihood of diet texture less than solid food (Normalcy of Diet score < 50) with low, medium and high volumes of high dose radiation (PTV Gy70)

Figure 4 represents probability curves over time, depicting the likelihood of diet texture less than solid food (Normalcy of Diet score < 50) with low, medium and high volumes of high dose radiation (PTV Gy70). There is an increased probability of poor outcome with advancing time, and higher radiation volume.

Xerostomia was prevalent but seemed to remain stable in later time intervals. There was no association between xerostomia and increased radiation dose to the parotid glands as determined by MD (mean dose) parotid on the radiation plans.

## Discussion

This is a single institution population-based consecutive case series including patients in Manitoba presenting with nasopharyngeal carcinoma from years 2008 to 2016 and treated curatively with IMRT. Compared to current literature, it is one of the most robust longitudinal studies available [12, 13]. Further strengthening this study is the prospective collection of validated functional outcome data obtained from patients at regular intervals by a dedicated Speech and Language Pathologist, with a high level of retention and compliance to long term data collection. Oncologic outcomes were similar to the reported survival in the AJCC 8th edition [13].

The study objectives were: provide longitudinal data on upper aerodigestive tract function and late complications following IMRT for nasopharyngeal carcinoma, and elucidate factors that might predict a worse outcome. The hypotheses were: (1) Despite advances such as IMRT, radiation will cause significant functional decline and late complications that often progress or arise years after treatment. (2) Larger radiation volume will be associated with poorer outcomes.

The longitudinal functional course of 42 patients has been successfully elucidated, and it appears as though late effects of radiation are indeed still common despite the use of IMRT. The late effects progress many years following treatment as seen through the regression modelling. Our data set was too small to support a robust multivariable analysis in search of independent predictors of worse outcome. However, the incremental increase in worse swallowing seen in patients treated with higher radiation volumes, and the shape of the probability curves (Fig. 4, function over time) demonstrating increasing dysfunction with time, suggest that radiation volume is major determinant of function.

PSS-HN outcomes suggested acute deterioration in the first 3 months, consistent with acute effects of treatment, followed by a period of recovery. Data from later follow-ups suggests functional decline due to new or advancing late effects of treatment ≥ 5 years post-treatment. These findings reflect acute treatment-related inflammation seen immediately following radiation, followed by accommodation and ultimately the late effects of radiation including tissue ischemia and fibrosis [14, 15]. Larger radiation volume predicted worse outcomes in all PSS-HN subscales; Eating in Public, Understandability of Speech, and Oral Diet Texture. The relationship between radiation volume, speech and swallowing has been previously described [16–19]. Both speech and swallowing require complex coordination of sensory and motor structures at high risk of radiation induced fibrosis [17, 18, 20]. Dose dependent injury to the constrictor, cricopharyngeal, and interarytenoid muscles results in long-term swallowing impairment, while atrophy, fibrosis, and loss of mucous cells within the larynx [21–24]. The data from VHI-10 and Dysphagia questionnaire are consistent with the PSS-HN outcomes, with a progressive worsening in symptoms noted from 24 months onwards. RBOHMS outcomes also showed an increase in need for feeding tube after 36 months follow-up with a rate of enteral feeding of 11% at ≥ 5 years.

An important component of this study is the data collected on late side effects of radiation. There was a correlation between time post treatment and episodes of aspiration pneumonia, with at least one death attributed to aspiration pneumonia. The etiology of aspiration pneumonia is likely due to a combination of dysphagia, xerostomia, and changes in sensation. Hypoglossal nerve dysfunction and trismus have also implicated in aspiration events and occurred at high rates in our population [25–28]. The incidence rate of hypoglossal nerve impairment was 18% at ≥ 5 years post-treatment, which is consistent with that in the literature; rates vary between 5 and 15%, depending on the time period assessed [29–32]. Importantly, there were no patients with reported hypoglossal nerve weakness at 3 months post treatment. The latency period of CNP onset is reported between 5 and 8 years, reportedly due to radiation-induced microvascular injury and resultant neural ischemia and demyelination [29, 33–35].

Trismus is another common sequela of radiation treatment, resulting from fibrosis to the temporomandibular joints and muscles of mastication [36, 37]. The location of NPC makes these structures particularly vulnerable. A 24% increase in the incidence rate of trismus has been reported with every additional 10 Gy of radiation to the pterygoid muscles [27]. This study identified high rates of trismus, with 40% of patients describing some element of reduced mouth opening 5 years following treatment. Only 5% reported symptoms at the initial 3-month follow-up. Muscle fibrosis and contracture has been reported progress at a rate of 2–4% loss of interincisal open per month shortly following radiation, ultimately resulting in a 30–35% reduction in mean interincisal open at 4 years [36, 38–40] A similar process affects all muscle and soft tissue included in the treatment field and explains the deterioration of neck flexion and increased rates of muscle spasms found in our population.

The main limitation of this study was the small group of patients. Despite this being one of the more robust studies of its kind, the numbers are far too small to support a robust multivariable analysis to clearly delineate predictors of poor functional outcome. Although this study has good retention of study subjects for late follow-up, even longer follow-up of 1–2 decades is required to clearly delineate the true extent of late treatment related damage to these patients. This study was primarily focused on speech, swallowing, and neuromuscular side effects relating to the upper aerodigestive tract and neck. There are many other late effects that must be considered when care for these patient: both very specific anatomic and physiologic issues such as middle and inner ear function, and the much broader measures and determinants of overall quality of life. Additionally, some of the 97 treated patients were excluded from the analysis as they declined regular participation in the evaluations. One possible cause is that these patients did not perceive themselves to be functionally impaired and therefore did not seek SLP follow-up. The study results therefore may be biased towards more severely affected patients. Future studies will be required to generate longer term data, and explore these other quality of life related issues.

## Conclusion

The findings in our study support the hypotheses that radiation late effects are still common and progressive after treatment of NPC, despite the advances in radiation delivery. Higher radiation volumes were associated with a greater decline in speech and swallowing. These effects need to be considered when planning treatment and advising patients regarding their long-term outcomes. These complications have implications for patients’ quality of life and overall survival, and support the life-long involvement of an allied health team in their care. Dedicated studies extending greater than 5 years should be undertaken to document and mitigate the enduring effects of radiation. Neoadjuvant chemotherapy may allow for reduced radiotherapy volumes, with the potential for reduced toxicity.

## Supplementary Information

**Additional file 1: Table S3.** Regression models for functional outcome data.

## Data Availability

The datasets used and/or analysed during the current study are available from the corresponding author on reasonable request.

## Abbreviations

IMRT: Intensity-modulated radiotherapy
NPC: Nasopharyngeal carcinoma
SLP: Speech and language pathologist
AJCC: American Committee on Cancer
MCR: Manitoba Cancer Registry
ICD-10-CA: International Classification of Diseases 10th revision for Canada
ICF: International Classification on Functioning, Disability, and Health
KPS: Karnofsky Performance Status scale
ECOG: Eastern Cooperative Oncology Group
PSS-HN: Performance Status Scale for Head and Neck Cancer Patients
RBHOMS: Royal Brisbane Hospital Outcome Measure for Swallowing
VHI-10: Voice Handicap Index-10
mESAS: Modified Edmonton Symptom Assessment Scale
GTVp: Gross tumour volume
GTVn: Gross nodal volume
PTV70: Planning target volume receiving 70 Gy
PTV59: Planning target volume receiving 59 Gy
PTVall: Sum of all target volumes receiving a therapeutic dose of radiation
MD parotid L, R: Mean dose to the left and right parotid
OS: Overall survival
DFS: Disease-free survival
DSS: Disease specific survival
VFSS: Videofluoroscopic swallow study
MBS: Modified barium swallow
CNP: Cranial nerve palsy
