# Supplementary material for: Longitudinal functional outcomes and late effects of radiation following treatment of nasopharyngeal carcinoma: secondary analysis of a prospective cohort study
Source: J Otolaryngol Head Neck Surg. 2022 Nov 8;51:41. doi: 10.1186/s40463-022-00593-7 (PMC9641961; doi:10.1186/s40463-022-00593-7)
Supplement: Supplementary file 1 — Additional file 1: Table S3. Regression models for functional outcome data. [file 40463_2022_593_MOESM1_ESM.docx]

Table 3.

| **Logistic mixed model predicting eating in public (predicting under 100: reference = 100)** | | | | |  |
| --- | --- | --- | --- | --- | --- |
|  |  |  | **OR** | **95% CI** | **p** |
| Treatment | Chemoradiation |  | 1.16 | 0.24 - 5.56 | 0.856 |
|  | Radiation |  | 1 |  |  |
| Stage | III-IV |  | 2.8 | 0.67 - 11.79 | 0.16 |
|  | I-II |  | 1 |  |  |
| GTVp volume | / 10 |  | 1.15 | 0.98 - 1.35 | 0.091 |
| PTV 70Gy | / 100 |  | 1.44 | 0.97 - 2.14 | 0.074 |
| PTV all | / 100 |  | 1.05 | 0.85 - 1.29 | 0.677 |
| Age | / 10 years |  | 1.49 | 0.98 - 2.25 | 0.061 |
| Gender | Female |  | 1.48 | 0.50 - 4.38 | 0.481 |
|  | Male |  | 1 |  |  |

| **Logistic mixed model predicting understandability of speech (predicting under 100: reference = 100)** | | | | |  |
| --- | --- | --- | --- | --- | --- |
|  |  |  | **OR** | **95% CI** | **p** |
| Treatment | Chemoradiation |  | / | / | / |
|  | Radiation |  | 1 |  |  |
| Stage | III-IV |  | 3.10 | 0.74-12.92 | 0.12 |
|  | I-II |  | 1 |  |  |
| GTVp volume | / 10 |  | 1.28 | 1.08 - 1.52 | 0.004 |
| PTV 70Gy | / 100 |  | 1.88 | 1.21 - 2.94 | 0.005 |
| PTV all | / 100 |  | 1.53 | 1.15 - 2.02 | 0.003 |
| Age | / 10 years |  | 1.00 | 0.65 - 1.55 | 0.996 |
| Gender | Female |  | 0.37 | 0.08 - 1.62 | 0.187 |
|  | Male |  | 1 |  |  |

| **Logistic mixed model predicting normalcy of diet (predicting 50 and under: reference = 60 - 100)** | | | | |  |
| --- | --- | --- | --- | --- | --- |
|  |  |  | **OR** | **95% CI** | **p** |
| Treatment | Chemoradiation |  | 2.20 | 0.61 - 7.93 | 0.227 |
|  | Radiation |  | 1 |  |  |
| Stage | III-IV |  | 4.20 | 1.40 - 12.64 | 0.011 |
|  | I-II |  | 1 |  |  |
| GTVp volume | / 10 |  | 1.07 | 0.89 - 1.27 | 0.466 |
| PTV 70Gy | / 100 |  | 1.55 | 1.02 - 2.36 | 0.041 |
| PTV all | / 100 |  | 1.16 | 0.94 - 1.43 | 0.164 |
| Age | / 10 years |  | 0.84 | 0.58 - 1.23 | 0.381 |
| Gender | Female |  | 0.78 | 0.27 - 2.29 | 0.651 |
|  | Male |  | 1 |  |  |

| **Logistic mixed model predicting RBHOMS (predicting under 8: reference = 8 - 10)** | | | | |  |
| --- | --- | --- | --- | --- | --- |
|  |  |  | **OR** | **95% CI** | **p** |
| Treatment | Chemoradiation |  | 2.38 | 0.30 - 18.92 | 0.411 |
|  | Radiation |  | 1 |  |  |
| Stage | III-IV |  | 1.81 | 0.44 - 7.47 | 0.411 |
|  | I-II |  | 1 |  |  |
| GTVp volume | / 10 |  | 1.13 | 0.95 - 1.34 | 0.168 |
| PTV 70Gy | / 100 |  | 1.50 | 0.94 - 2.39 | 0.087 |
| PTV all | / 100 |  | 1.18 | 0.92 - 1.50 | 0.192 |
| Age | / 10 years |  | 1.45 | 0.87 - 2.43 | 0.151 |
| Gender | Female |  | 1.65 | 0.45 - 6.04 | 0.447 |
|  | Male |  | 1 |  |  |

| **Logistic mixed model predicting Voice Handicap Index (predicting above 0: reference = 0)** | | | | |  |
| --- | --- | --- | --- | --- | --- |
|  |  |  | **OR** | **95% CI** | **p** |
| Treatment | Chemoradiation |  | 1.73 | 0.46 - 6.57 | 0.421 |
|  | Radiation |  | 1 |  |  |
| Stage | III-IV |  | 1.63 | 0.50 - 5.27 | 0.416 |
|  | I-II |  | 1 |  |  |
| GTVp volume | / 10 |  | 1.06 | 0.89 - 1.26 | 0.504 |
| PTV 70Gy | / 100 |  | 1.40 | 0.93 - 2.11 | 0.103 |
| PTV all | / 100 |  | 1.15 | 0.91 - 1.43 | 0.237 |
| Age | / 10 years |  | 1.11 | 0.75 - 1.65 | 0.589 |
| Gender | Female |  | 0.60 | 0.18 - 1.98 | 0.406 |
|  | Male |  | 1 |  |  |

| **Logistic mixed model predicting Karnofsky (predicting under 90: reference = 90-100)** | | | | |  |
| --- | --- | --- | --- | --- | --- |
|  |  |  | **OR** | **95% CI** | **p** |
| Treatment | Chemoradiation |  | 0.30 | 0.04 - 2.14 | 0.228 |
|  | Radiation |  | 1 |  |  |
| Stage | III-IV |  | 1.86 | 0 - 137709.97 | 0.914 |
|  | I-II |  | 1 |  |  |
| GTVp volume | / 10 |  | / | / | / |
| PTV 70Gy | / 100 |  | / | / | / |
| PTV all | / 100 |  | / | / | / |
| Age | / 10 years |  | 2.02 | 1.03 - 3.97 | 0.041 |
| Gender | Female |  | 2.74 | 0.63 - 11.87 | 0.178 |
|  | Male |  | 1 |  |  |

| **Logistic mixed model predicting ECOG (predicting above 0: reference = 0)** | | | | |  |
| --- | --- | --- | --- | --- | --- |
|  |  |  | **OR** | **95% CI** | **p** |
| Treatment | Chemoradiation |  | 0.36 | 0.09 - 1.42 | 0.146 |
|  | Radiation |  | 1 |  |  |
| Stage | III-IV |  | 1.69 | 0.51 - 5.61 | 0.391 |
|  | I-II |  | 1 |  |  |
| GTVp volume | / 10 |  | / | / | / |
| PTV 70Gy | / 100 |  | 1.02 | 0.68 - 1.54 | 0.926 |
| PTV all | / 100 |  | 0.91 | 0.72 - 1.14 | 0.405 |
| Age | / 10 years |  | 1.83 | 1.01 - 3.31 | 0.046 |
| Gender | Female |  | 2.17 | 0.68 - 6.91 | 0.188 |
|  | Male |  | 1 |  |  |

| **Clustered quantile regression model predicting dysphagia** | | | | |  |
| --- | --- | --- | --- | --- | --- |
|  |  |  | **Median**  **Difference** | **95% CI** | **p** |
| Treatment | Chemoradiation |  | -1.00 | -4.37 - 2.37 | 0.562 |
|  | Radiation |  | (reference) |  |  |
| Stage | III-IV |  | 2.00 | -0.74 - 4.74 | 0.156 |
|  | I-II |  | (reference) |  |  |
| GTVp volume | / 10 |  | 0.00 | -0.27 - 0.27 | 0.998 |
| PTV 70Gy | / 100 |  | 0.40 | -0.51 - 1.31 | 0.391 |
| PTV all | / 100 |  | 0.22 | -0.42 - 0.86 | 0.495 |
| Age | / 10 years |  | -0.19 | -1.29 - 0.92 | 0.741 |
| Gender | Female |  | 0.97 | -2.22 - 4.15 | 0.553 |
|  | Male |  | (reference) |  |  |

| **Clustered quantile regression model predicting xerostomia** | | | | |  |
| --- | --- | --- | --- | --- | --- |
|  |  |  | **Median**  **Difference** | **95% CI** | **p** |
| Treatment | Chemoradiation |  | -1.86 | -6.32 - 2.60 | 0.415 |
|  | Radiation |  | (reference) |  |  |
| Stage | III-IV |  | -0.24 | -2.94 - 2.47 | 0.863 |
|  | I-II |  | (reference) |  |  |
| GTVp volume | / 10 |  | -0.09 | -0.38 - 0.20 | 0.552 |
| PTV 70Gy | / 100 |  | -0.04 | -0.94 - 0.86 | 0.933 |
| PTV all | / 100 |  | -0.07 | -0.58 - 0.43 | 0.783 |
| MD parotid L | / 1000 |  | 0.18 | -0.68 - 1.03 | 0.684 |
| MD parotid R | / 1000 |  | 0.02 | -1.49 - 1.52 | 0.981 |
| MD parotid L+R/2 | / 1000 |  | 0.28 | -0.95 - 1.52 | 0.654 |
| Age | / 10 years |  | -0.02 | -1.36 - 1.32 | 0.979 |
| Gender | Female |  | 0.03 | -2.75 - 2.81 | 0.983 |
|  | Male |  | (reference) |  |  |
